# Identification of a glycolysis-related gene signature for predicting prognosis in patients with hepatocellular carcinoma

**DOI:** 10.1186/s12885-022-09209-9

**Published:** 2022-02-05

**Authors:** Junjie Kong, Guangsheng Yu, Wei Si, Guangbing Li, Jiawei Chai, Yong Liu, Jun Liu

**Affiliations:** 1 Department of Liver Transplantation and Hepatobiliary Surgery, Shandong Provincial Hospital, Cheeloo College of Medicine, Shandong University, Jinan, 250021 Shandong Province China; 2 Department of Liver Transplantation and Hepatobiliary Surgery, Shandong Provincial Hospital Affiliated to Shandong First Medical University, Jinan, 250021 Shandong China; 3 Department of Breast and Thyroid Surgery, Shandong Maternity and Child Care Hospital, Jinan, 250014 Shandong Province China

**Keywords:** Hepatocellular carcinoma, Glycolysis, Prognosis, GSEA, Gene signature, Tumor mutational burden, TCGA

## Abstract

**Background:**

Hepatocellular carcinoma (HCC) is the most common primary liver cancer in the world. Although great advances in HCC diagnosis and treatment have been achieved, due to the complicated mechanisms in tumor development and progression, the prognosis of HCC is still dismal. Recent studies have revealed that the Warburg effect is related to the development, progression and treatment of various cancers; however, there have been a few explorations of the relationship between glycolysis and HCC prognosis.

**Methods:**

mRNA expression profiling was downloaded from public databases. Gene set enrichment analysis (GSEA) was used to explore glycolysis-related genes (GRGs), and the LASSO method and Cox regression analysis were used to identify GRGs related to HCC prognosis and to construct predictive models associated with overall survival (OS) and disease-free survival (DFS). The relationship between the predictive model and the tumor mutation burden (TMB) and tumor immune microenvironment (TIME) was explored. Finally, real-time PCR was used to validate the expression levels of the GRGs in clinical samples and different cell lines.

**Results:**

Five GRGs (ABCB6, ANKZF1, B3GAT3, KIF20A and STC2) were identified and used to construct gene signatures to predict HCC OS and DFS. Using the median value, HCC patients were divided into low- and high-risk groups. Patients in the high-risk group had worse OS/DFS than those in the low-risk group, were related to higher TMB and were associated with a higher rate of CD4+ memory T cells resting and CD4+ memory T cells activated. Finally, real-time PCR suggested that the five GRGs were all dysregulated in HCC samples compared to adjacent normal samples.

**Conclusions:**

We identified five GRGs associated with HCC prognosis and constructed two GRGs-related gene signatures to predict HCC OS and DFS. The findings in this study may contribute to the prediction of prognosis and promote HCC treatment.

**Supplementary Information:**

The online version contains supplementary material available at 10.1186/s12885-022-09209-9.

## Background

Hepatocellular carcinoma (HCC), as the most common primary liver cancer, causes nearly 78,000 deaths each year and is the fourth leading cause of cancer-related deaths in the world [1]. Although great advances have been achieved in HCC treatment, including progress in surgery, radiotherapy, chemotherapy and immunotherapy [2], the prognosis of HCC is still dismal, and it is estimated that the 5-year overall survival (OS) of HCC remains less than 20% [3]. The rapid development of molecular targeted therapy has provided a new choice for HCC treatment. Molecular biomarkers could contribute to patient survival improvement and optimize therapeutic strategies, and they could also be used in the diagnosis, prediction of prognosis and prediction of response to systemic therapies [4]. However, since HCC is a neoplasm with complicated mechanisms in development, progression and recurrence, the biomarkers existing are far from sufficient, and there is still a requirement to explore more molecular biomarkers for the diagnosis and treatment of HCC.

Recently, advancements in the next-generation sequencing technology have led to a great increase in the identification of molecular biomarkers [5, 6]. New biomarkers related to tumor development, progression and prognosis could be identified using bioinformatic methods, which might contribute to revealing the complex biological processes related to cancers and could be potential therapeutic targets. For instance, using online databases, Chen et al. [7]. identified three immune-related genes that have a potential role in immune checkpoint inhibitor therapy for head and neck squamous cell carcinoma. In addition, using The Cancer Genome Atlas (TCGA) database, methylation profiling suggested that several biomarkers including DET (amplification), WNT signalling (CTNNB1 mutation) and IDH1 (mutation) were potential therapeutic targets for HCC [8].

Glycolysis is a series of metabolic processes that universally exist in living cells and is essential for the metabolism of cells [9]. Recent studies revealed that “metabolic reprogramming” might be a hallmark of cancer [10], and glycolysis, as a crucial process in cell metabolism, could hardly absolve itself from the blame. Tumor cells exhibit a characteristic of increasing glycolysis and obtaining their energy needs using this metabolic pathway, which is also known as the Warburg effect [11]. The Warburg effect could contribute cancer cells to a progression advantage [12] and was reported to be related to the development, progression and treatment of various cancers [13]. In HCC, recent studies reported that biomarkers involved in the Warburg effect could be therapeutic targets [14]. Using bioinformatic methods, several glycolysis-related genes (GRGs) were identified to be related to the diagnosis and prognosis of HCC [15, 16]. However, the biochemical and molecular mechanisms of the Warburg effect are complicated, and more GRGs need to be explored.

Tumor mutational burden (TMB), defined as the number of mutations per megabase in tumor cells, is a predictive biomarker of immunotherapy response [17]. It was reported that tumors with higher TMB tend to have more immunogenic neoantigens, making them recognized by immune cells [18]. In some tumor types, TMB is helpful to select patients who could benefit from immunotherapy [17]. A recent study revealed that there was a positive correlation between glycolysis and TMB in some types of cancers [19]. In addition, the tumor immune microenvironment (TIME) refers to a complex microenvironment that mainly contains immune cells and immune-related molecules [20]. The TIME plays crucial roles in immunotherapeutic responsiveness in various cancers [21]. It was reported that there was a correlation between glycolysis and TIME. On the one hand, glycolysis could increase tumor immunity in some types of cancers. For example, glycolysis could increase PD-L1 expression on tumor cells [22]. Furthermore, similar to cancer cells, immune cells can induce glycolysis after they are active.

In this study, using data obtained from TCGA, Gene Expression Omnibus (GEO) and International Cancer Genome Consortium Japan (ICGC) databases, we identified five GRGs related to HCC prognosis. Afterwards, two predictive models were constructed to predict OS and disease-free survival (DFS) for HCC. Finally, the relationship between glycolysis and tumor mutation burden (TMB) and the tumor immune microenvironment (TIME) were explored. These results may provide new insight for the prediction of HCC prognosis and treatment.

## Methods

### Dataset collection

First, the transcriptome profiling data (FPKM values) of HCC, which included 374 HCC tissues and 50 nontumor tissues, were downloaded from the TCGA database (https://www.cancer.gov/). The related clinical information was obtained from the online database “cBioPortal” (http://www.cbioportal.org/). Second, using the keyword “hepatocellular carcinoma”, we searched for datasets from the GEO database (https://www.ncbi.nlm.nih.gov/gds/), and the organism’s parameter was selected “*Homo sapiens*”. The selection criteria were as follows: 1) datasets with more than 200 HCC samples; 2) datasets with available mRNA expression information; and 3) datasets with comparisons between HCC samples and nontumor liver samples. Three GEO profiles, GSE25097, GSE36376 and GSE14520, were selected; among them, the GSE14520 dataset contained prognostic information and was used for survival analysis. Third, the mRNA expression dataset with a clinical file (LIRI-JP) was downloaded from the ICGC (International Cancer Genome Consortium) database (https://icgc.org/), which contained 442 tumor samples and 224 normal samples. Afterwards, a mRNA pool was constructed through overlapping genes among the TCGA, ICGC and GEO datasets. Furthermore, the TCGA dataset was used as the training dataset, and the others were used as the validation dataset. This project was approved by the Ethics Committee of Shandong Provincial Hospital Affiliated to Shandong First Medical University and was performed in accordance with the Declaration of Helsinki. Each participant provided written informed consent.

### Gene set enrichment analysis (GSEA) and identification of GRGs

After the primary screening of patients in the TCGA dataset, thirty-three patients were excluded, including three without related clinical information, three with fibrolamellar carcinoma, eight with combined hepatocellular-cholangiocarcinoma, and seventeen who received R1 and R2 resection, and a total of 343 HCC patients were included in the GSEA analysis. GSEA 4.1.0 [23, 24] was used to identify glycolysis-related gene sets that significantly differed between tumor and nontumor samples. Six glycolysis-related gene sets (Table S1) were downloaded from the Molecular Signatures Database (https://www.gsea-msigdb.org/gsea/msigdb/index.jsp). For GSEA analysis, the parameter for gene sets permutations was set 1000 times, and a false discovery rate (FDR) < 0.05 was considered significant. Three glycolysis-related gene sets were found to significantly differ between HCC and nontumor samples, and 255 GRGs were identified from the three gene sets and were included in the following analysis. Finally, we made an overlap between the 255 GRGs and the mRNA pool constructed above, and a total of 203 GRGs were identified in all five datasets.

To avoid the batch effects, using the “sva” R package, the expression levels of GRGs in TCGA and ICGC datasets were standardized. Afterwards, using the “limma” R package, we identified GRGs differentially expressed between tumor and nontumor samples in the TCGA dataset, with an adjusted *p* value < 0.01 and an absolute log2-fold change > 1 used as selection criteria. Finally, 83 GRGs were identified as dysregulated between tumor and nontumor samples and were included in the survival analysis.

### Construction and validation of GRGs-related gene signature for HCC patients

In the training set, univariate, LASSO and multivariate Cox regression analyses were used to identify GRGs related to OS. Using the “survival” R package, univariate Cox regression was conducted to make a primary selection of GRGs related to OS, and those with *p* < 0.05 were considered potential candidates. Afterwards, using the “glmnet” R package, LASSO Cox regression [25] was conducted to further select the potential candidates. The dataset was subsampled for 1000 iterations, and GRGs with occurrence frequencies of more than 900 were selected for the following analysis. Finally, a step multivariate Cox regression analysis was used to identify GRGs related to OS and to construct a predictive gene signature. Using the regression coefficient (β) of multivariate Cox regression analysis, a risk score for each patient was calculated using the following formula: risk score = (β_mRNA1_ * expression level of mRNA1) + (β_mRNA2_ * expression level of mRNA2) + …. + (β_mRNAn_ * expression level of mRNAn). Using the median value of the risk score, HCC patients were divided into two groups: high- and low-risk groups. The differences in clinicopathological traits between the two groups were analysed. Kaplan–Meier analysis and receiver operating characteristic (ROC) curves were employed to evaluate the predictive ability of the gene signature.

### Independent prognostic role of the GRGs-related gene signature and establishment and validation of a predictive nomogram

Univariate and multivariate Cox regression analyses were used to explore independent prognostic factors for HCC in the TCGA dataset. For univariate analysis, factors with a *p* < 0.05 were considered statistically significant and were included in multivariate analysis. Based on the results of multivariate analysis, a predictive nomogram was established using the “rms” R package to predict the 1-, 3- and 5-year OS for HCC [26]. Calibration curves were used to assess the performance of the nomogram. The C-index and time-dependent ROC curves were used to evaluate the discrimination of the nomogram. Furthermore, using the “rmda” R package, decision curve analysis (DCA) was used to assess the clinical net benefit of the nomogram [27].

### Estimation of tumor mutational burden (TMB) and immune cell type fractions

The somatic mutation data were downloaded from TCGA GDC Data Portal. Using a Perl script, the gene mutation rate and the TMB in different risk groups were calculated based on the somatic mutation data, and the “maftools” R package was used to visualize the results. Furthermore, the TMB difference between the low- and high-risk groups was compared using the Mann–Whitney U test, and the impact of TMB on HCC prognosis was evaluated using Kaplan–Meier analysis. CIBERSORT analysis (https://cibersortx.stanford.edu/) was used to estimate the fraction of the 22 subtype immune cells in each TCGA HCC sample [28]. The Tumor Immune Estimation Resource (TIMER, version 2.0, http://timer.cistrome.org/) online database was used to evaluate the impact of GRGs on the infiltration of different immune cells [29].

### Gene set enrichment analysis

To identify signaling pathways regulated by the GRG-related gene signature, GSEA was used, and “c2.cp.kegg.v7.2.symbols.gmt” was employed as the reference gene set. Terms with a *p* value < 0.05 were considered statistically significant, and the results were visualized using the “ggplot2” R package.

### Validation of the expression and prognostic ability and genetic alterations of GRGs

First, the expression levels of GRGs between tumor and nontumor samples were compared in training and validation datasets, different cell lines and the Shandong Provincial Hospital patient dataset. Afterwards, Kaplan–Meier analysis was used to evaluate the predictive ability of GRGs. The Human Protein Atlas database (https://www.proteinatlas.org/) was used to evaluate the expression of encoded proteins of the five GRGs. Using the “corrplot” R package, the correlation of expression among GRGs was explored. Finally, using the “cBioPortal” database, the genetic alterations of the GRGs were analysed.

### Cell lines

LO_2_, HepG2, MHCC97-H, SK-HEP1 and Bel-7402 cell lines were purchased from the BeNa Culture Collection (Beijing, China). LO_2_ and Bel-7402 cell lines were cultured in RPMI-1640 (Gibco, NYC, USA) containing 10% fetal bovine serum (Gibco, NYC, USA), and the other cell lines were cultured in DMEM (Gibco, NYC, USA) containing 10% fetal bovine serum. All cell lines were culture at 37 °C and 5% CO_2_.

### Real-time PCR analysis

To validate the dysregulation of the five GRGs in in vitro experiments, 10 pairs of samples with the pathological diagnosis of HCC and adjacent nontumor samples were prospectively collected from our hospital. Total RNA was extracted from HCC samples, paired adjacent normal samples and cell lines using TRIzol reagent (Invitrogen, Eugene, OR, USA) following the manufacturer’s instructions. Complementary DNA (cDNA) was synthesized using 1 μg of total RNA and HiScript III RT SuperMix (Vazyme, Nanjing, China). RT–PCR was performed using ChamQ Universal SYBR qPCR Master Mix (Vazyme, Nanjing, China), which was executed by a QuantStudio 3 Real-Time PCR System. The following primers were used in RT–PCR: ABCB6 F: GCCTCATTGTGTTCCTGTGC and R: CACTTCGTAACTCTCGGCGT; ANKZF1 F: CTCTGGCCGGTCTTTGTTCT and R: CACACGCTGTAGCTCTTGGA; B3GAT3 F: GGGACCCAAGGAGTCGTCTA and R: CTGTGTGGAAGCCCACTACC; KIF20A F: AAGGGCAGAACTGGCTCATC and R: GCAAGGGCTTCAGATCAGGT; STC2 F: TGAAATGTAAGGCCCACGCT and R: TTGAGGTAGCATTCCCGCTG.

### Statistical analysis

Continuous variables are displayed as the mean ± standard deviation (SD), and categorical variables are shown as numbers (n) and percentages (%). The Mann–Whitney U test was used to compare the difference between continuous variables, and the categorical variables were compared by using the chi-squared test or Fisher’s test. Kaplan–Meier analysis and the log-rank test were employed to analyse OS and DFS. Cox regression analysis was used to identify independent factors for prognosis, and factors with *p* < 0.05 in univariate analysis were included in multivariate analysis. The hazard ratio (HR) and 95% confidence interval (CI) were recorded. R software (Version 4.0.3) was used for the statistical analysis. All tests were two sides, and *p* < 0.05 was considered statistically significant.

## Results

### Identification of GRGs in HCC

**Fig. 1:**
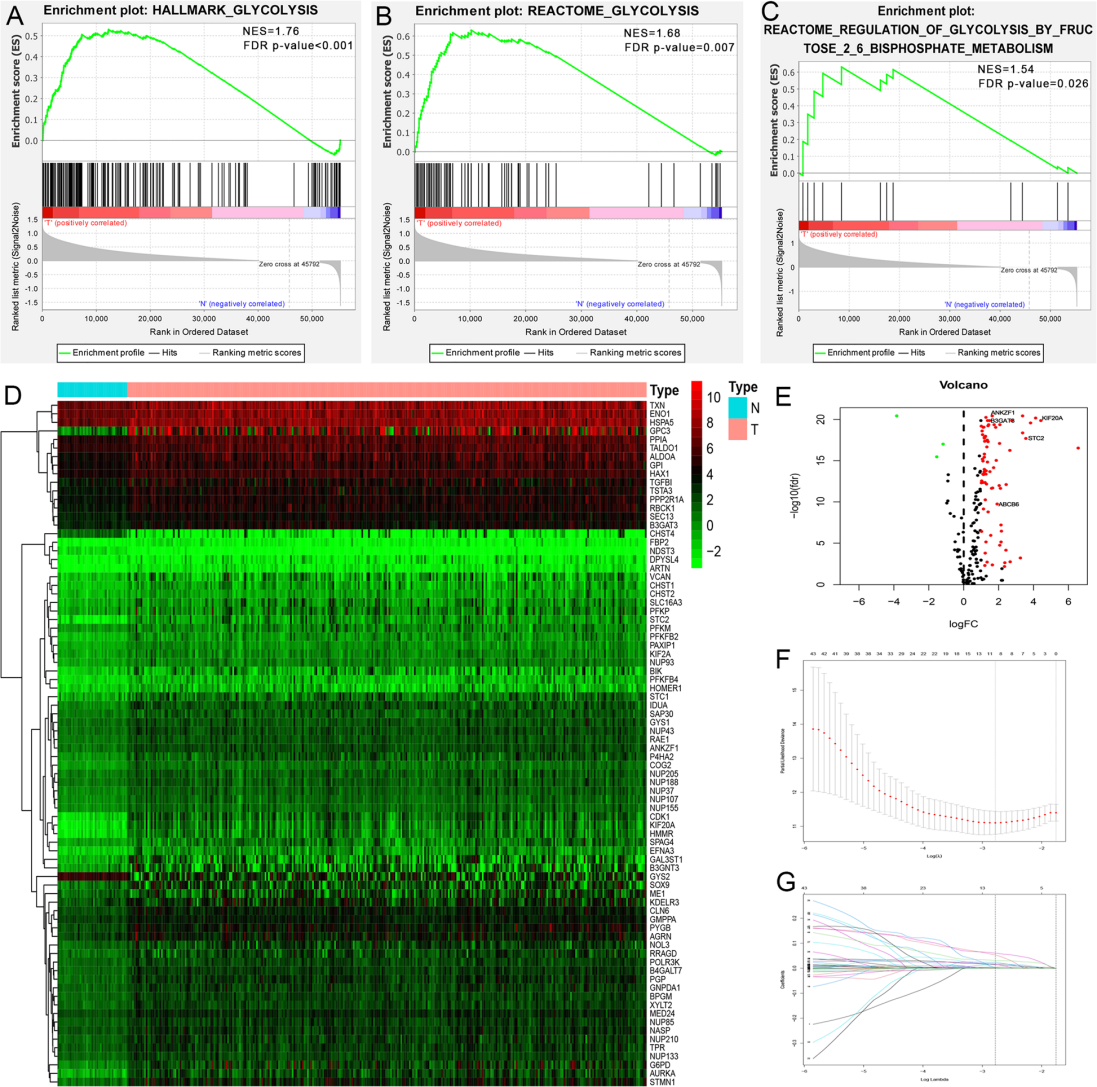
GSEA, heatmap, volcano and LASSO Cox regression identified the GRGs associated with prognosis in HCC. **A**, **B**, **C** Enrichment plots of three glycolysis-related gene sets between HCC and normal samples identified by GSEA. **D**, **E** Gene expression levels in the TCGA dataset. **F**, **G** LASSO Cox regression analysis for the exploration of GRGs related to HCC prognosis. GSEA, Gene set enrichment analysis; HCC, hepatocellular carcinoma; GRGs, glycolysis-related genes

GSEA revealed that three of the six glycolysis-related gene sets, including hallmark glycolysis (*p* < 0.001), Reactome glycolysis (*p* = 0.007) and glycolysis and Reactome regulation of glycolysis by fructose 2,6-bisphosphate metabolism (*p* = 0.026), were significantly enriched in tumor samples (Fig. 1A-C). Subsequently, 255 GRGs were identified in the TCGA dataset. After an overlapping analysis with the mRNA pool, a total of 203 GRGs were identified in all five datasets. Finally, 83 GRGs were differentially expressed in HCC samples compared with nontumor samples in the TCGA dataset (Fig. 1D-E). The overall study design and workflow was shown in Fig. S1.

### Identification of GRGs related to OS and construction of a gene signature for prediction of OS

After removing patients with unknown survival information, those with less than 3 months of survival and those without clinicopathological information (including sex, age at diagnosis, TNM stage, grade, weight and height), a total of 273 HCC patients in the TCGA dataset were included in the survival analysis. We first performed univariate Cox regression analysis to explore GRGs related to OS, and 50 GRGs were found to be statistically significant (*p* < 0.05). Afterwards, LASSO Cox regression analysis was performed, and the optimal tuning parameters related to the minimum generalization error were determined from 10-fold cross-validation. Ten genes were identified with a repetition frequency greater than 900 times in 1000 substitution samplings (Fig. 1F-G). Finally, step multivariate Cox regression analysis was performed, and 5 GRGs, ABCB6, ANKZF1, B3GAT3, KIF20A and STC2 (Table S2), were found to be independently associated with OS. Using the β of multivariate analysis, we constructed a prognostic gene signature containing the five GRGs to predict the OS for each HCC patient as follows: risk score = (0.134 * expression level of ABCB6) + (0.072 * expression level of ANKZF1) + (0.031 * expression level of B3GAT3) + (0.150 * expression level of KIF20A) + (0.034 * expression level of STC2).

**Fig. 2:**
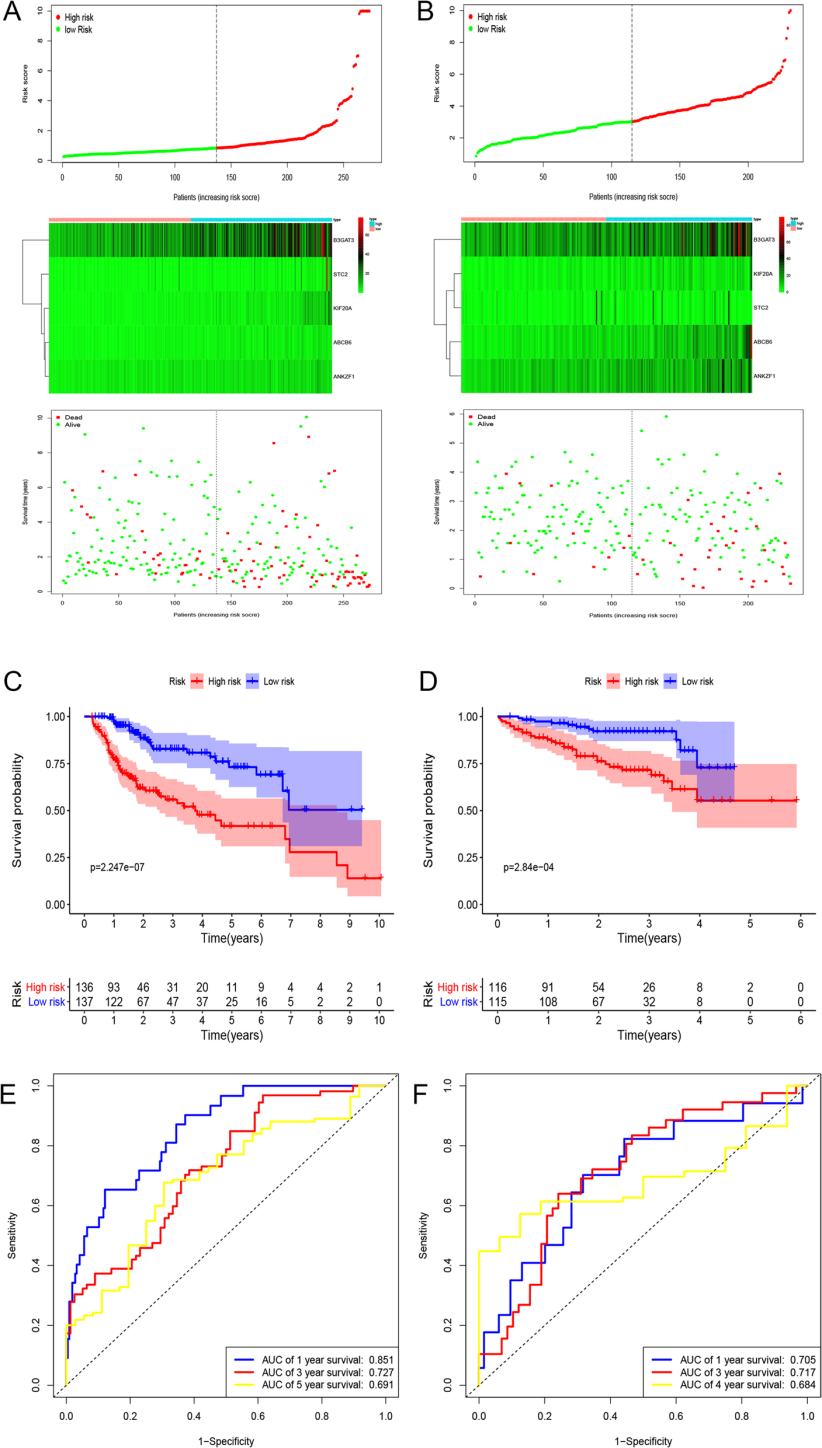
Risk score distribution, Kaplan-Meier analysis and time-dependent ROC analysis of a prognostic model for OS in HCC patient cohort from training (**A**, **D**, **G**) and validation datasets. **A**, **B** The distribution of risk score and survival status corresponding to the expression of each gene. **C**, **D** Kaplan-Meier analysis suggested that patients in high-risk group had shorter OS than those in low-risk group. **E**, **F** Time-dependent ROC curves to assess the performance of gene signature. ROC, receiver operating characteristic; OS, overall survival; HCC, hepatocellular carcinoma; ICGC, International Cancer Genome Consortium Japan

Using the median value of the risk score, we divided HCC patients in the TCGA dataset into two groups: low- and high-risk groups. Figure 2A shows the distribution of the risk score and survival status corresponding to the expression of each gene. The Kaplan–Meier analysis showed that patients in the high-risk group had significantly poorer OS than those in the low-risk group (Fig. 2C). The areas under the curve (AUCs) of the time-dependent ROC curves at 1, 3 and 5 years were 0.851, 0.727 and 0.691, respectively, which suggests a high sensitivity and specificity of the gene signature for predicting OS (Fig. 2E). The relationship between the gene signature and clinicopathological factors in the TCGA dataset is shown in Table S3. We also validated the performance of gene We also validated the performance of the gene signature in predicting OS in the validation set. The distribution of risk score and survival status corresponding to gene expression levels are shown in Fig. 2B. The Kaplan–Meier analysis also suggested that patients in the high-risk group had a worse OS than those in the low-risk group in the ICGC dataset (Fig. 2D). The AUCs of the ROC curves for the prediction of 1-, 3- and 5-year survival in the ICGC dataset were 0.601, 0.638 and 0.649, respectively (Fig. 2F).

### Construction and validation of a nomogram for OS prediction cooperating with GRGs-related gene signature

**Fig. 3:**
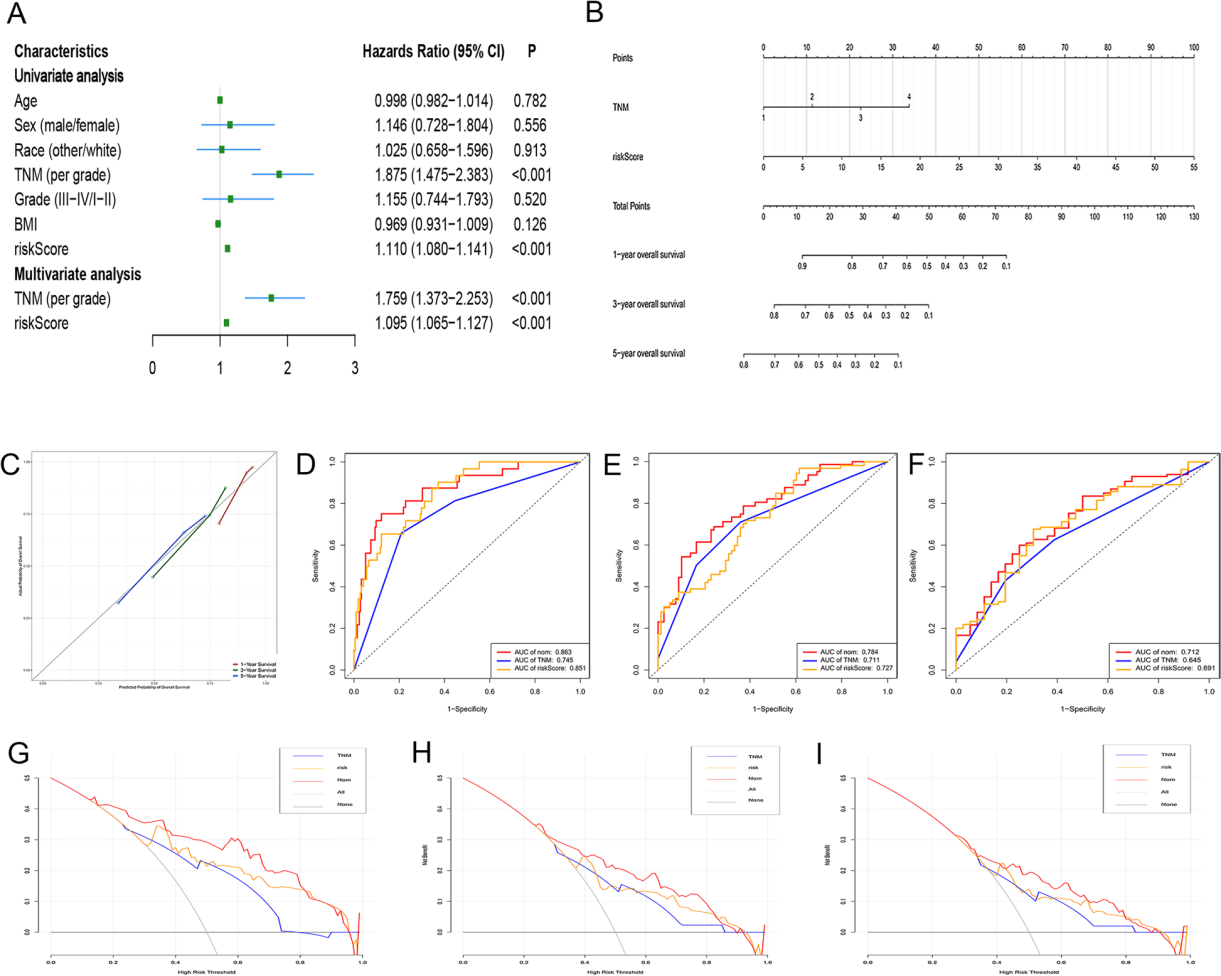
Establishment and validation of a nomogram to predict OS for HCC. **A** Univariate and multivariate Cox regression analysis to explore factors independently associated with OS. **B** The nomogram for predicting the 1-, 3- and 5-year OS for HCC. **C** Calibration curves of the nomogram for predicting the 1-, 3- and 5-year OS for HCC. **D**, **E**, **F** ROC curves to evaluate the performance of the nomogram in predicting the 1-, 3- and 5-year OS, respectively. **G**, **H**, **I** DCA to assess the clinical net benefit of the nomogram in predicting the 1-, 3- and 5-year OS, respectively. OS, overall survival; HCC, hepatocellular carcinoma; ROC, receiver operating characteristic; DCA, decision curve analysis

Cox regression analysis was used to explore whether the gene signature was an independent prognostic factor when clinicopathological traits (including age, sex, grade, TNM stage and BMI) were included in the analysis. The results of the Cox regression analysis are shown in Fig. 3A. We found that the GRG-related gene signature and TNM stage were independently associated with OS. Based on the results of multivariate analysis, a nomogram containing the gene signature and TNM stage was established to predict the 1-, 3- and 5-year OS for HCC (Fig. 3B). The C-index of the nomogram was 0.749 (95% CI, 0.633-0.865). The calibration curves of the nomogram for predicting the 1-, 3- and 5-year OS suggested a high consistency between actual observations and prediction of OS (Fig. 3C).

We then performed ROC curve analysis to assess the discrimination ability of the nomogram, and the results showed that the AUCs for the prediction of 1-, 3- and 5-year OS were 0.863, 0.784 and 0.712, respectively, which were higher than those of using the gene signature or TNM stage alone (Fig. 3D-F). Afterwards, DCA analysis was performed to evaluate the value of the nomogram in guiding clinical decision making, and the results showed that the nomogram could obtain better net benefit than that of using the gene signature or TNM stage alone (Fig. 3G-I).

### Construction and validation of a GRGs-related gene signature for predicting DFS

**Fig. 4:**
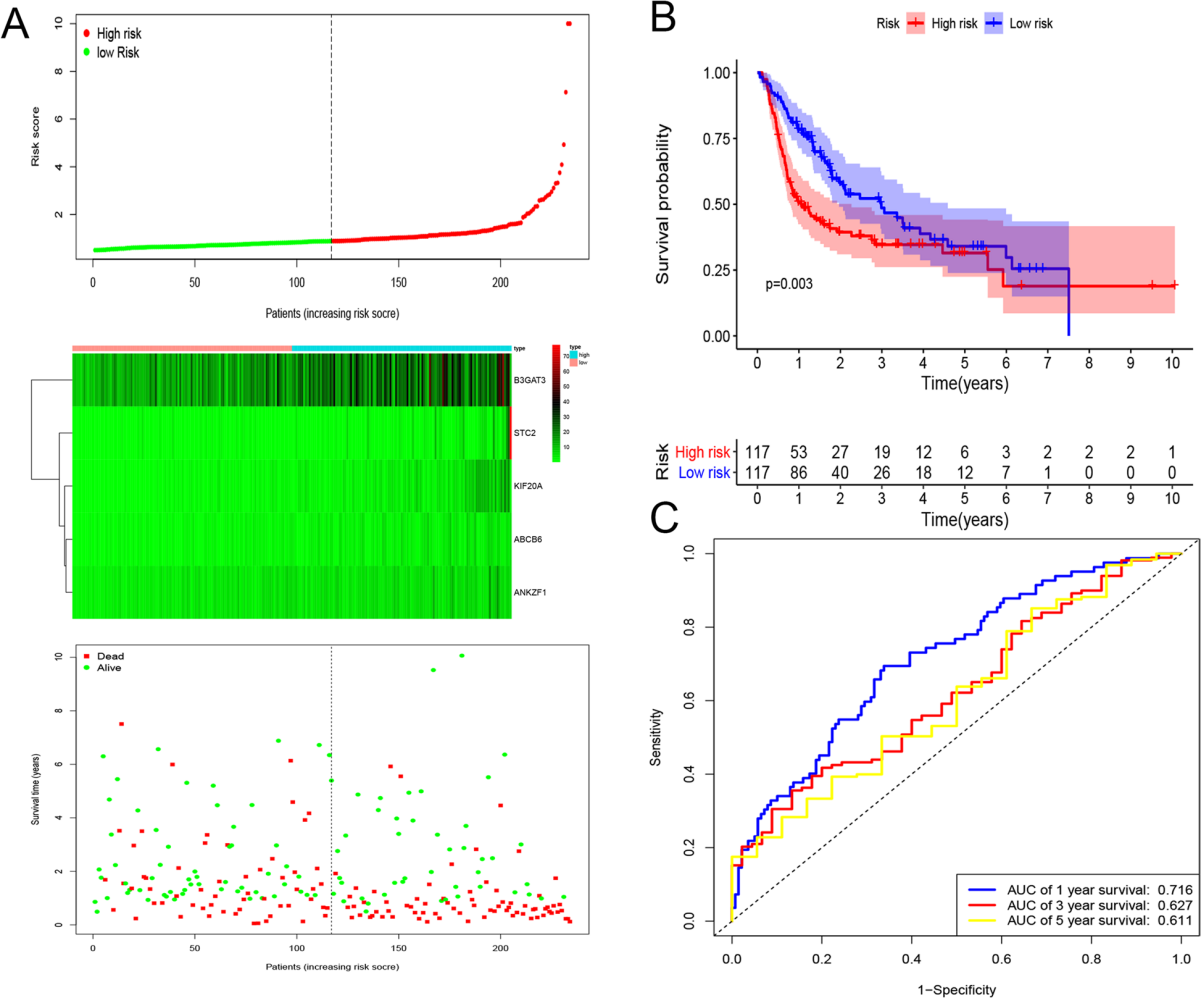
Risk score distribution, Kaplan-Meier analysis and time-dependent ROC analysis of a prognostic model for DFS. **A** The distribution of risk score and survival status corresponding to the expression of each gene. **B** Kaplan-Meier analysis suggested that patients in high-risk group had shorter DFS than those in low-risk group. **C** Time-dependent ROC curves to assess the performance of gene signature. ROC, receiver operating characteristic; DFS, disease-free survival; HCC, hepatocellular carcinoma

After removing patients with less than 3 months of survival, with unknown DFS status and with unknown clinicopathological information (including age, TNM stage, grade, height and weight), a total of 234 HCC patients in the TCGA dataset were included in the analysis. Using the five GRGs, a gene signature was constructed based on the results of multivariate analysis: risk score = (0.063 * expression level of ABCB6) + (0.046 * expression level of ANKZF1) + (0.014 * expression level of B3GAT3) + (0.084 * expression level of KIF20A) + (0.059 * expression level of STC2). Using the median value of the risk score, HCC patients were divided into low- and high-risk groups. The distribution of the risk score and DFS status corresponding to gene expression is shown in Fig. 4A. The Kaplan–Meier analysis suggested that patients in the high-risk group had a higher recurrence rate than those in the low-risk group (*p* = 0.003, Fig. 4B). The ROC curve analysis showed that the gene signature had a relatively high accuracy for predicting DFS, with 1-, 3- and 5-AUC values of 0.716, 0.627 and 0.611, respectively (Fig. 4C).

### Construction and validation of a nomogram for predicting DFS in cooperation GRGs-related gene signature

**Fig. 5:**
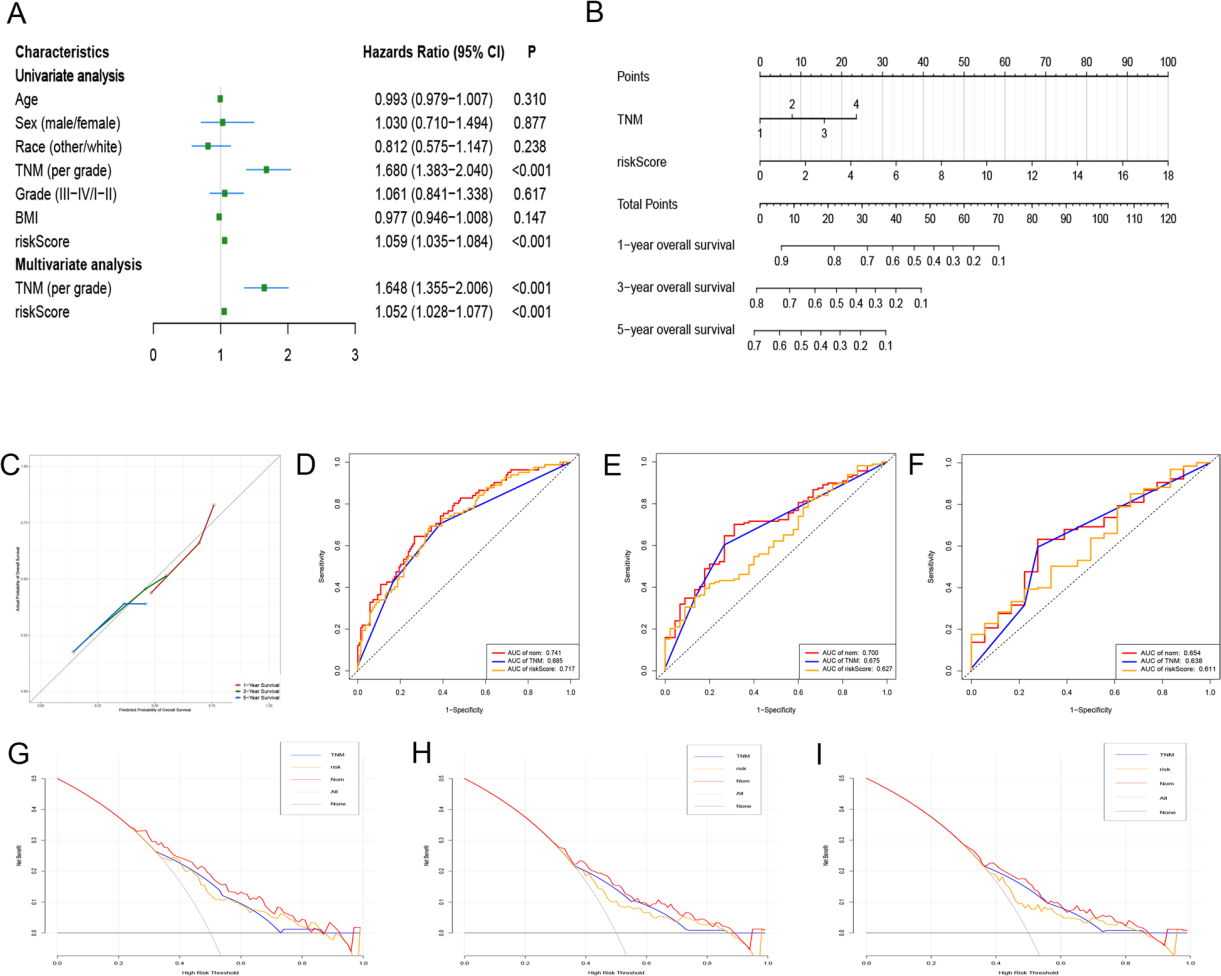
Establishment and validation of a nomogram to predict DFS for HCC. **A** Univariate and multivariate Cox regression analysis to explore factors independently associated with DFS. **B** The nomogram for predicting the 1-, 3- and 5-year DFS for HCC. **C** Calibration curves of the nomogram for predicting the 1-, 3- and 5-year DFS for HCC. **D**, **E**, **F** ROC curves to evaluate the performance of the nomogram in predicting the 1-, 3- and 5-year DFS, respectively. **G**, **H**, **I** DCA to assess the clinical net benefit of the nomogram in predicting the 1-, 3- and 5-year DFS, respectively. DFS, disease-free survival; HCC, hepatocellular carcinoma; ROC, receiver operating characteristic; DCA, decision curve analysis

Univariate and multivariate Cox regression analyses were performed, and two factors (gene signature and TNM stage) were found to be independently associated with DFS (Fig. 5A). Afterwards, a nomogram was constructed integrating the two factors to predict 1-, 3- and 5-DFS for HCC (Fig. 5B). The C-index of the nomogram was 0.695 (95% CI 0.579-0.811). The calibration curves of the nomogram suggested that it performed well (Fig. 5C). The AUCs of the ROC curve analysis for 1-, 3- and 5-year recurrence were 0.741, 0.700 and 0.654, respectively, which displayed a larger AUC than that of the gene signature or TNM stage alone and showed that the nomogram had a high discrimination ability (Fig. 5D-F). Finally, DCA also showed that the nomogram had a higher clinical net benefit than that of using the gene signature or TNM stage alone (Fig. 5G-I).

### Comparison of the TMB and TIME of HCC patients between low- and high-risk groups and GSEA

**Fig. 6:**
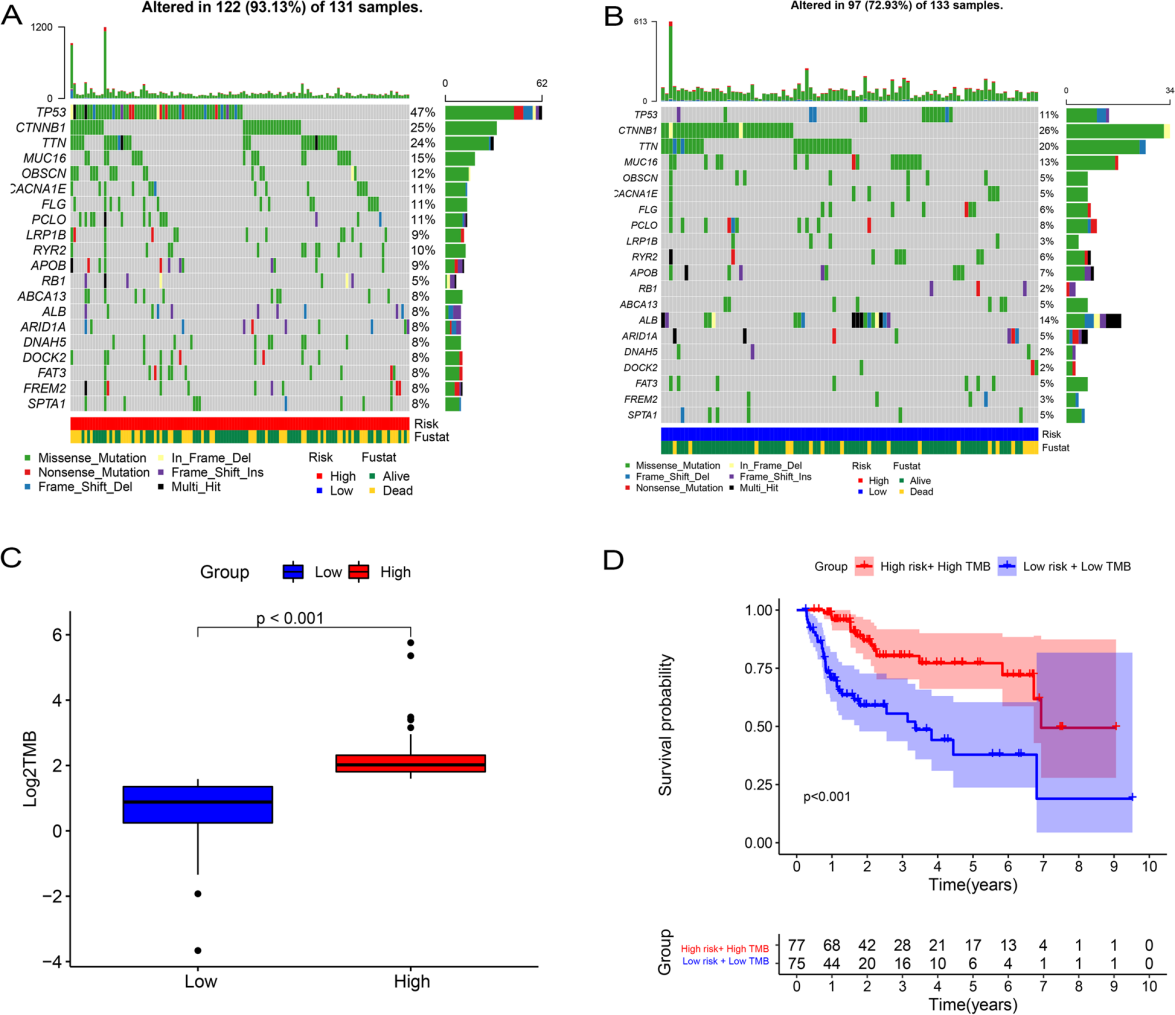
Correlation between gene signature and TMB and the predictive performance of TMB on OS. **A**, **B** The differences in TMB in somatic cells in high- and low-risk groups. **C** Patients in high-risk group had higher TMB than those in low-risk group. **D** Patients in high-risk group with high TMB had a worse OS than those in low-risk group with low TMB. TMB, tumor mutational burden; OS, overall survival

**Fig. 7:**
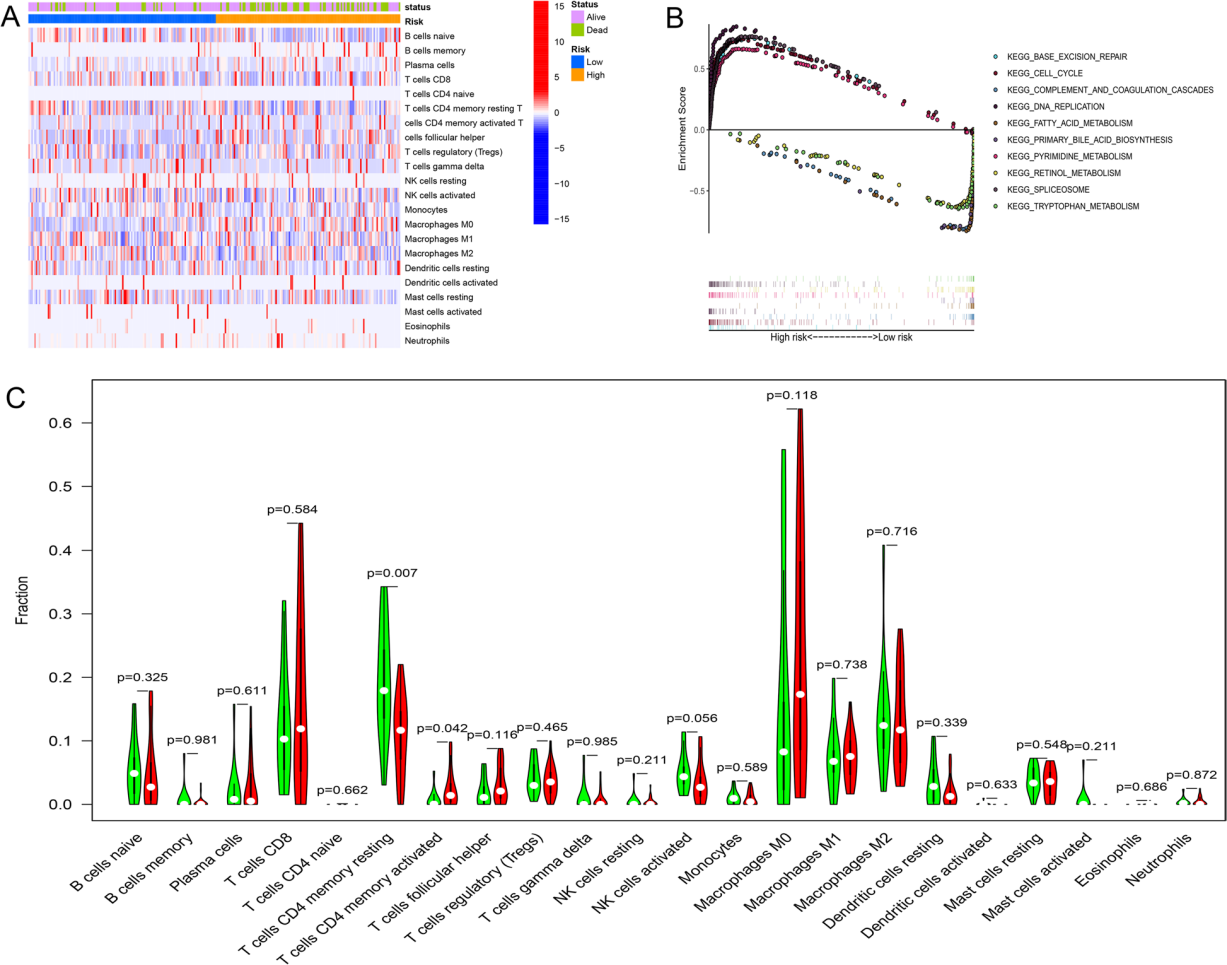
The landscape of immune infiltration and GSEA analysis for HCC patients in different risk groups. **A**, **C** The relationships between gene signature and immune infiltration of 22 immune cell types in HCC patients. **B** GSEA analysis for HCC patients in low- and high-risk groups. GSEA, Gene set enrichment analysis; HCC, hepatocellular carcinoma

Considering the positive relationship between glycolysis and TMB and TIME, we made a further comparison of TMB and TIME between the low- and high-risk groups. Figure 6A and B show the differences in TMB in somatic cells in the high- and low-risk groups. As displayed in Fig. 6C and D, we found that patients in the high-risk group had higher TMB than those in the low-risk group, and patients in the high-risk group with high TMB had a worse OS than those in the low-risk group with low TMB (*p* < 0.001). Figure 7A shows the distributions of 22 tumor-infiltrating immune cell types obtained from 273 HCC patients which were selected in the GSEA analysis. Furthermore, as shown in Fig. 7C, patients in the high-risk group had a higher rate of CD4+ memory T cell resting and CD4+ memory T cell activation.

GSEA was conducted to identify the biological processes and signaling pathways in which the GRGs were enriched. The results showed that GRGs in the high-risk group were mainly enriched in base excision repair, the cell cycle, DNA replication and pyrimidine metabolism. GRGs in the low-risk group were significantly enriched in complement and coagulation cascades, fatty acid metabolism, primary bile acid biosynthesis, retinol metabolism, spliceosome and tryptophan metabolism (Fig. 7B).

### Validation of the expression, prognostic ability and genetic alterations of GRGs

**Fig. 8:**
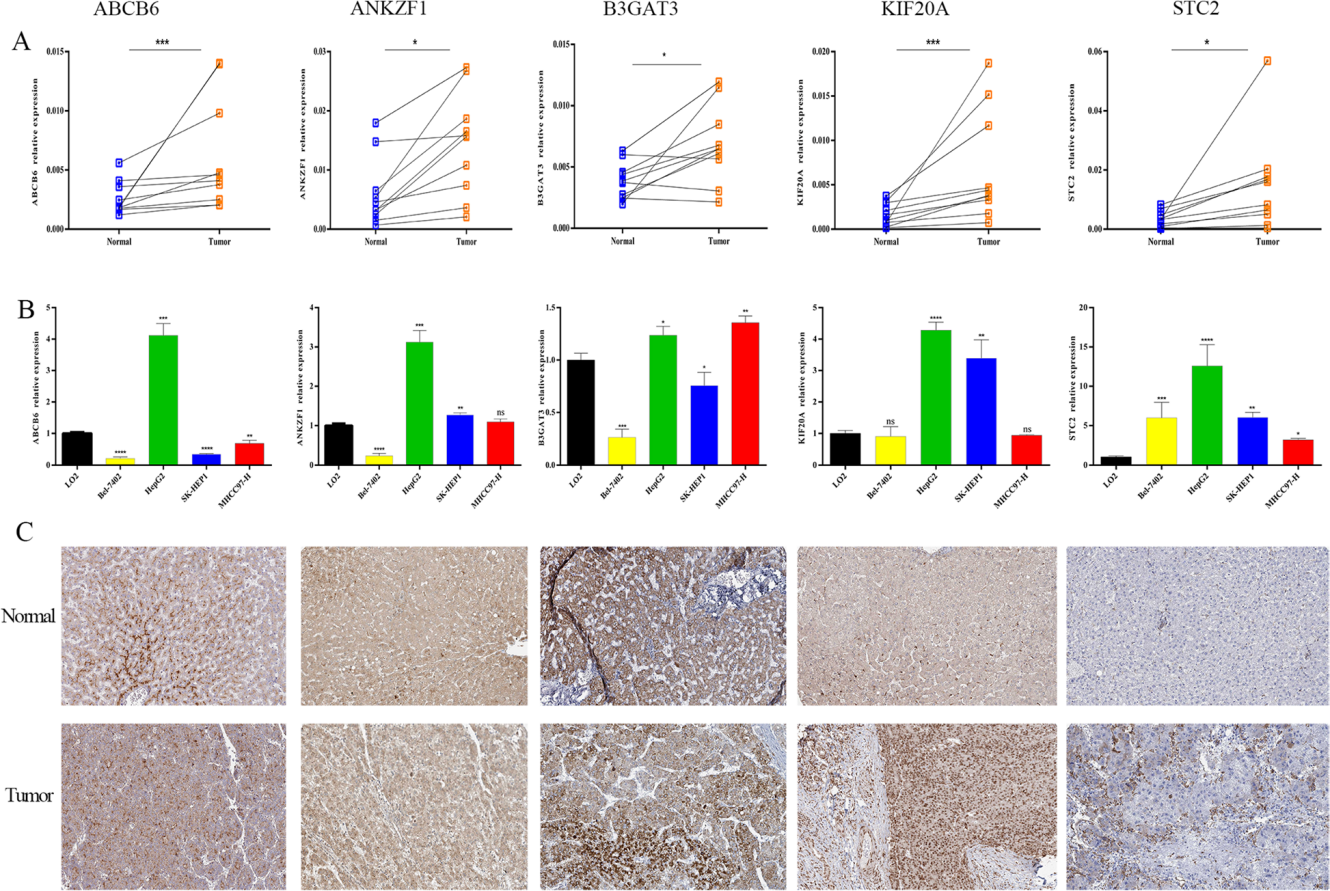
Expression validation of the five GRGs. **A** Real-time PCR analysis of the mRNA expression levels of the five GRGs in HCC and normal samples in Shandong Provincial Hospital patient cohort. **B** Real-time PCR analysis of the mRNA expression levels of the five GRGs in different cell lines. **C** Expression patterns of the five GRGs in HCC and normal samples. GRGs, glycolsis-related genes; HCC, hepatocellular carcinoma

To validate the expression levels of GRGs between tumor and nontumor samples, we first explored the expression of the five GRGs in public datasets, and the results showed that the five GRGs were significantly dysregulated in HCC samples compared with normal samples (Fig. S2). We also explored the expression level of the five GRGs in our own samples, and the results showed that the five GRGs were highly expressed in tumor tissues compared with nontumor samples (Fig. 8A). Meanwhile, the expression profiles of the five GRGs in hepatoma cell lines and normal cell lines are shown in Fig. 8B. Using the Human Protein Altas database, we also explored the immunohistochemistry results of the five GRGs. The patient immunochemistry data are shown in Table S3, and we found that the intensity of the encoded proteins of the five GRGs in HCC samples was stronger than that in nontumor samples (Fig. 8C). We also tried to explore the relationship of expression levels among the five GRGs, and the results suggested that there is a strong correlation between ANKZF1 and KIF20A as well as ABCB6 (Fig. S3).

**Fig. 9:**
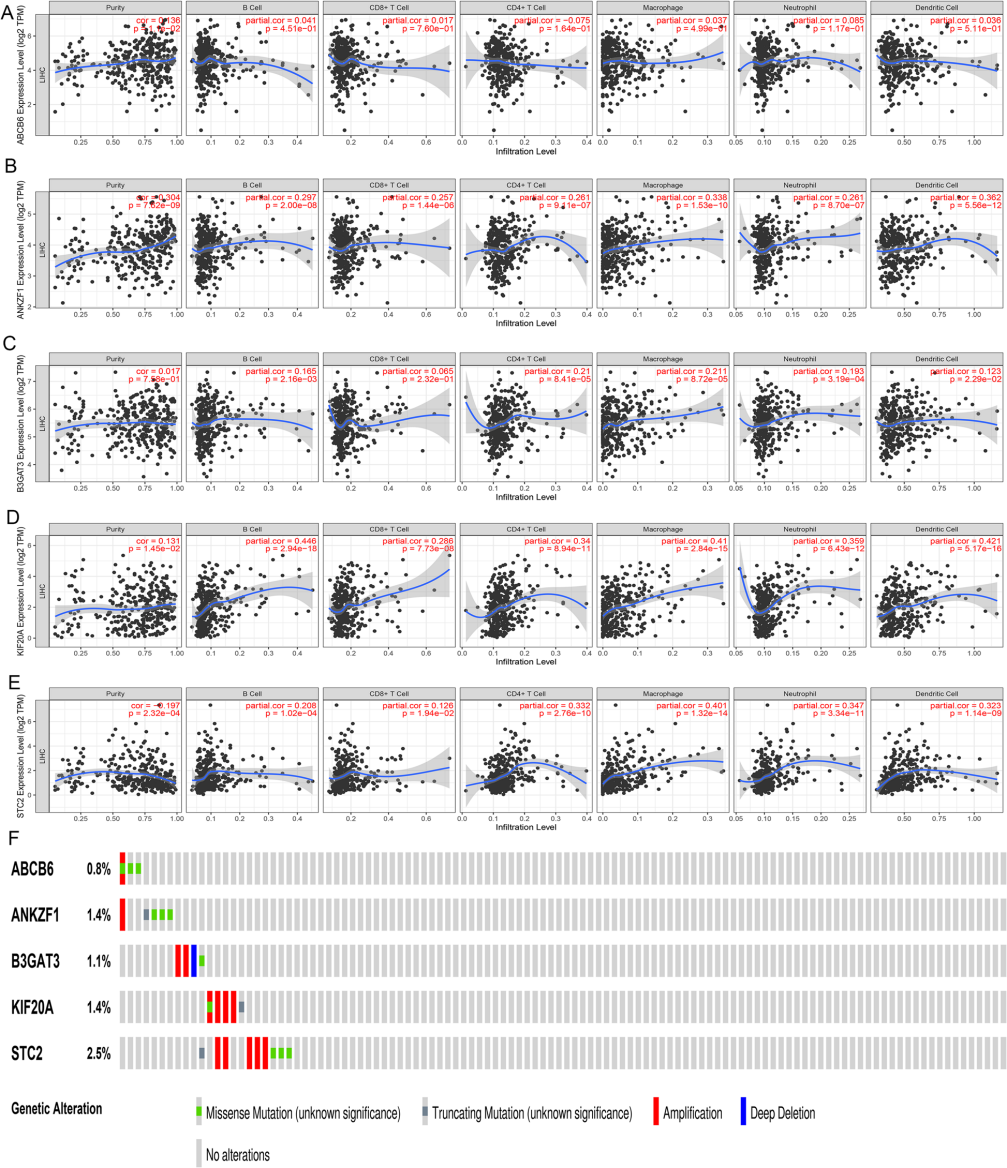
Correlations between GRGs and the density of the immune infiltrate and the genetic alterations analysis for the five GRGs. **A-E** The influence of the expression of ABCB6 (**A**), ANKZF1 (**B**), B3GAT3 (**C**), KIF20A (**D**) and STC2 (**E**) on infiltration by different immune cells. **F** Genetic alteration analysis of the five GRGs. GRGs, glycolysis-related genes; HCC, hepatocellular carcinoma

Using the TIMER online database, the relationship between the five GRGs and immune infiltration was explored, which is shown in Fig. 9A-E. In addition, using the survival data in the TCGA dataset, we attempted to explore the prognostic ability for each GRG. Kaplan–Meier analysis showed that the high expression of the five GRGs was significantly associated with poorer OS (*P* < 0.05, Fig. S4A-E), while those with high expression of the five GRGs also had poorer DFS (*P* < 0.05, Fig. S4F-J). Finally, we evaluated the genetic alterations of the five GRGs, and the results suggested that the proportions of the genetic alterations for the five GRGs were 0.8, 1.4, 1.1, 1.4 and 2.5% (Fig. 9F).

## Discussion

HCC, as a medical problem for society, still has a dismal prognosis. There is an urgent need to explore core molecular biomarkers that are related to HCC development and prognosis and could be used as therapeutic targets. In this study, using data obtained from TCGA, GEO and ICGC databases, we aimed to explore GRGs related to the prognosis of HCC using bioinformatic methods, Cox regression analysis and LASSO methods. Five GRGs (ABCB6, ANKZF1, B3GAT3, KIF20A and STC2) were dysregulated in tumor samples and were independently associated with the prognosis of HCC. Afterwards, two gene signatures containing the five GRGs were established to predict OS and DFS for HCC. The calibration curves and ROC curves showed that the gene signatures had good predictive efficiencies. Finally, combining the gene signatures and clinicopathological traits, two predictive nomograms were constructed, and the C-index, calibration curves, ROC curves and DCA analysis all indicated the good performance of the nomograms in predicting the prognosis of HCC.

Five GRGs were finally upregulated in HCC samples and were independently associated with the prognosis of HCC. ABCB6, a member of the lysosomal ATP-binding cassette (ABC) transporters, is widely expressed in many tissues, including liver, heart and skeletal muscles [30]. The main function of ABCB6 is regulating porphyrin biosynthesis; recently, it has been reported to be associated with multidrug resistance and the development and progression of various cancers [31–33]. In HCC, Polireddy et al. [34] discovered that ABCB6 was upregulated in tumor samples and was related to tumor development and progression by regulating the cell cycle. Furthermore, Tsunedomi et al. [35] proved that the dysregulation of ABCB6 was associated with intrahepatic recurrence and poor prognosis of HCC. However, due to the controversial role of ABCB6, the mechanisms of ABCB6 in HCC development, progression and recurrence remain unclear. Among the other four GRGs, ANKZF1 had the strongest correlation with ABCB6. ANKZF1, also known as ankyrin repeat and zinc-finger domain-containing 1, can combine with p97 and thus regulate the function of p97, including the cell cycle, apoptosis and autophagy [36]. The upregulation of ANKZF1 has been reported to be related to poor OS and DFS in colon cancer. Furthermore, Chen et al. [37] proved that ANKZF1 was a GRG related to the prognosis of colon adenocarcinoma. In HCC, the prognostic ability of ANKZF1 has not been reported, and further explorations are needed. Beta-1,3-glucuronyltransferase 3, encoded by the B3GAT3 gene, is crucial for proteoglycan (PG) biosynthesis [38]. Since PG could affect the cell cycle and was related to tumor cell invasion and metastasis [39, 40], the potential role of B3GAT3 in tumor development, progression and prognosis was explored [41]. Recently, Zhang et al. [42] found that B3GAT3 was upregulated in HCC samples, and the dysregulation of B3GAT3 was associated with poorer pathological characteristics, tumor invasion, migration and a more adverse prognosis. Unlike the three GRGs above, the potential role of KIF20A and STC2 in tumor development, progression and prognosis has been widely studied. Kinesin family member 20A (KIF20A), a member of the kinesin superfamily of proteins [43], plays a crucial role in cytokinesis [44]. A recent study found that KIF20A was upregulated in the lactate-enriched tumor microenvironment and could regulate microtubule dynamics and cell motility and thus contribute to cancer progression and metastasis [45]. In HCC, KIF20A was also discovered to be upregulated in tumor samples, which could contribute to tumor cell proliferation and was associated with poorer tumor characteristics and shorter OS and DFS [46, 47]. Stanniocalcin 2 (STC2) is associated with phosphate metabolism and glucose homeostasis [48, 49]. In addition, it was also found to be associated with angiogenesis, tumor progression and metastasis [48]. Previous studies showed that STC2 was upregulated in HCC samples, and in vitro experiments suggested that the dysregulation of STC2 could promote tumor cell proliferation and migration [48]. In addition, Cheng et al. [50] proved that upregulation of STC2 could mediate drug resistance in HCC cells, which provided a potential strategy for HCC treatment. In this study, GSEA showed that the GRG-related gene signature was associated with several cell cycle-related pathways, which was consistent with the functions of the five GRGs.

We further explored the relationship between the risk groups and TMB and TIME. TMB refers to the number of mutations per megabase in tumor cells [51], and it was reported that tumor cells with high TMB were more likely to contain neoantigens to be targets of immune cells; thus, TMB was regarded as a biomarker for predicting immunotherapy response and prognosis [18]. In this study, we found that high-risk group was related to higher TMB, and patients in high-risk group with high TMB had poorer OS than those in low-risk group with low TMB. In addition, the TIME is a complex microenvironment that mainly contains immune cells and immune-related molecules. TIME is closely related to tumor progression, prognosis and antitumor immunotherapy [52, 53]. We further found that patients in the high-risk group had a high rate of resting CD4+ memory T cells and activated CD4+ memory T cells. It was reported that memory CD4+ T cells were related to tumor growth and the effects of chemotherapy [54, 55]. Our results suggested that the TIME in the high-risk group might be associated with the poor prognosis of these patients. These findings suggested that HCC patients in the high-risk group might have a higher response to immune therapy than those in the low-risk group, which might provide new insight into HCC immunotherapy.

Apart from our study, several studies have been published to explore glycolysis-related genes (GRGs) associated with HCC prognosis [56, 57]. However, the methods used to screen GRGs varied in these studies. For example, in Hamaguchi’s study [56], they explored GRGs based on the overlap between the glycolysis module genes and their own pooled transcriptome data. In Lu’s study [57], they explored GRGs based on the expression level of glycolytic components (glucose transporter and glycolytic enzymes). Each TCGA sample obtained a glycolysis score, and these samples were divided into high and low glycolysis score groups. Afterwards, genes differentially expressed between the two groups were identified and regarded as GRGs. In our study, GSEA was used to identify GRGs: all glycolysis-related gene sets were obtained from the Molecular Signatures Database v4.0, and using GSEA, gene sets that significantly differed between HCC and nontumor samples were identified. Genes in these gene sets were regarded as the “primary” GRGs. Afterwards, using the “limma” R package and univariate, LASSO and multivariate Cox regression analyses, we finally obtained GRGs related to HCC prognosis. Obviously, the various methods used in different studies, combined with the heterogeneity in data processing and patient selection, could all cause the difference in identification of GRGs. In our study, using comprehensive bioinformatic analysis, we obtained five GRGs associated with HCC prognosis. We also performed external validation using HCC samples from GEO and ICGC datasets and our own data. All of these efforts could make our results more reliable.

Many gene signatures have been constructed to predict HCC patient survival [58–61]. Compared to these gene signatures, our gene signature had the following features: first, our gene signature was established based on five GRGs that were independently associated with HCC prognosis. Considering the vital role of glycolysis in tumor development and progression, our gene signature is surely to have a better predictive performance than the previous predictive models [58–61]; second, some of the previous gene signatures lacked external validation using other datasets [60], consequently, we used the ICGC-LIRI—JP as an external validation cohort to verify the results of this study, which showed that the gene signature had good predictive performance.

There are some limitations in this study. First, this is a study using public datasets for different patient cohorts, and the results could be somewhat heterogeneous in data processing and patient selection. Although we validated our gene signature in external datasets, prospective cohorts with more HCC patients are needed to validate our risk models. Second, we downloaded all available clinical information of TCGA patients from the online database; however, several important factors, such as AFP level, portal hypertension and postoperative complications, were missing, and the incorporation of these factors might greatly improve the predictive ability of our nomogram. Finally, the complicated interactions between GRGs and the TMB and TIME need further exploration.

## Conclusions

To summarize, we identified five GRGs related to survival between HCC and nontumor samples. Two prognostic models containing the five GRGs were constructed to predict OS and DFS for HCC, and the C-index, calibration curves, ROC analysis and DCA analysis all showed good performance of the two risk models. In addition, HCC patients in high-risk groups were also found to have higher proportions of TMB and immune cell infiltration, which may be more sensitive to immunotherapy.

## Supplementary Information

**Additional file 1: Figure S1.** The overall study design and workflow. HCC, hepatocellular carcinoma; ICGC, International Cancer Genome Consortium Japan; GSEA, Gene set enrichment analysis; GRGs, glycolysis-related genes; OS, overall survival; DFS, disease-free survival; ROC, receiver operating characteristic.

**Additional file 2: Figure S2.** Expression validation of the five GRGs in public datasets. (A) TCGA, (B) ICGC, (C) GSE 14520, (D) GSE 25097 and (E) GSE 36376 datasets. GRGs, glycolysis-related genes; ICGC, International Cancer Genome Consortium Japan.

**Additional file 3: Figure S3.** Correlation between the five glycolysis-related genes in the dataset from TCGA.)

**Additional file 4: Figure S4.** The predictive ability of the five GRGs on OS and DFS. (A-E) The relationship between the dysregulation of GRGs and OS in HCC. (F-J) The relationship between the dysregulation of GRGs and DFS in HCC. GRGs, glycolysis-related genes; HCC, hepatocellular carcinoma; OS, overall survival; DFS, disease-free survival.

**Additional file 5: Table S1.** Detailed information for six glycolysis-related gene sets.

**Additional file 6: Table S2.** Multivariate Cox regression analysis of GRGs independently associated with HCC prognosis.

**Additional file 7: Table S3.** Correlation between gene signature and clinicopathological factors.

**Additional file 8: Table S4.** Information of Immunohistochemistry in the Human Protein Atlas database.

## Data Availability

The datasets used and/or analyzed during the current study are available from the corresponding author on reasonable request.

## Abbreviations

HCC: Hepatocellular carcinoma
GSEA: Gene set enrichment analysis
GRGs: Glycolysis-related genes
OS: Overall survival
DFS: Disease-free survival
TMB: Tumor mutational burden
TCGA: The Cancer Genome Atlas
GEO: Gene Expression Omnibus
ICGC: International Cancer Genome Consortium Japan
TIME: Tumor immune microenvironment
FDR: False discovery rate
ROC: Receiver operating characteristic
DCA: Decision curve analysis
cDNA: Complementary DNA
SD: Standard deviation
HR: Hazard ratio
CI: Confidence interval
