# Supplementary figures and images for: Identification of a glycolysis-related gene signature for predicting prognosis in patients with hepatocellular carcinoma
Source: BMC Cancer. 2022 Feb 5;22:142. doi: 10.1186/s12885-022-09209-9 (PMC8817563; doi:10.1186/s12885-022-09209-9)

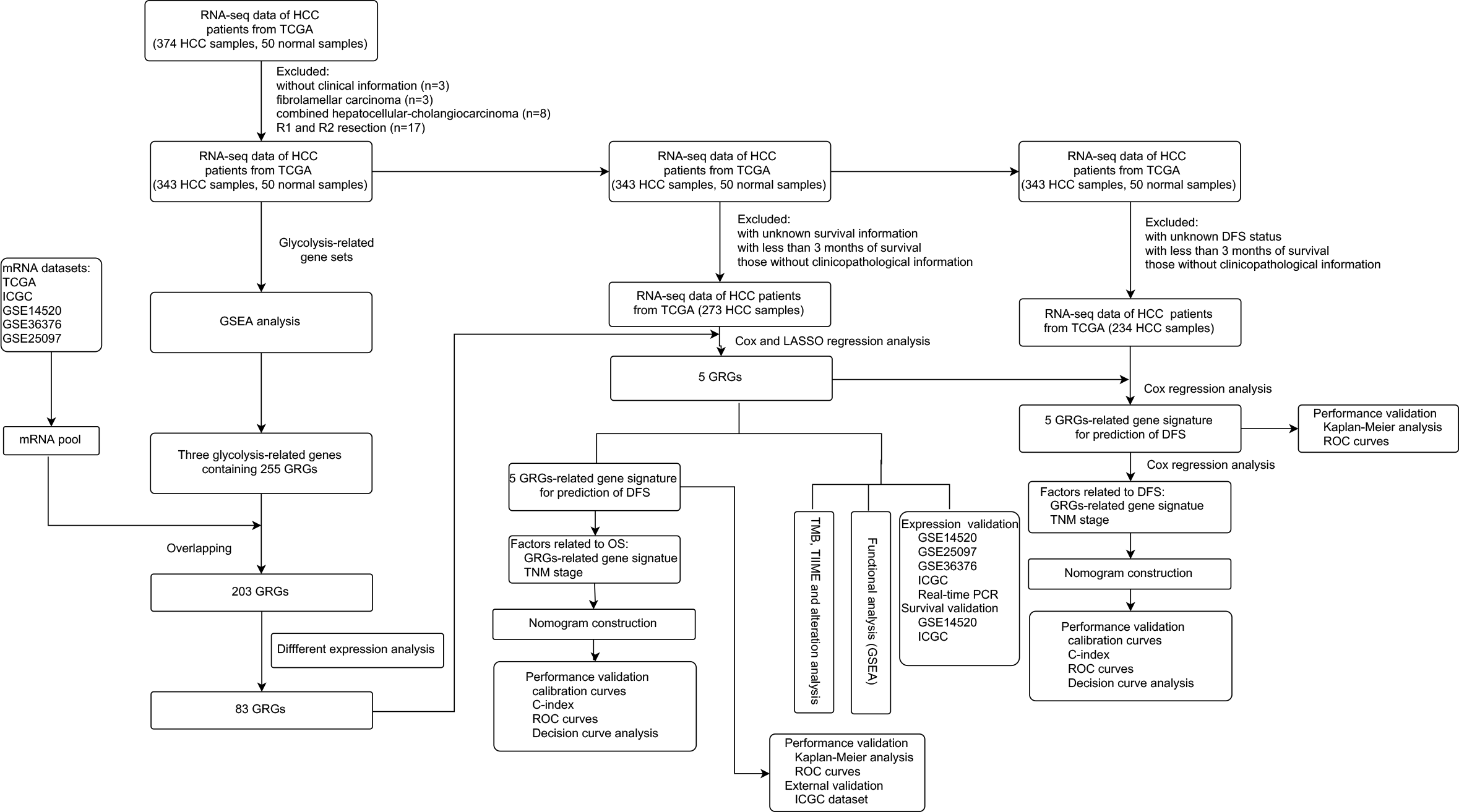

Supplement: Supplementary file 1 — Additional file 1: Figure S1. The overall study design and workflow. HCC, hepatocellular carcinoma; ICGC, International Cancer Genome Consortium Japan; GSEA, Gene set enrichment analysis; GRGs, glycolysis-related genes; OS, overall survival; DFS, disease-free survival; ROC, receiver operating characteristic. [file 12885_2022_9209_MOESM1_ESM.tif]

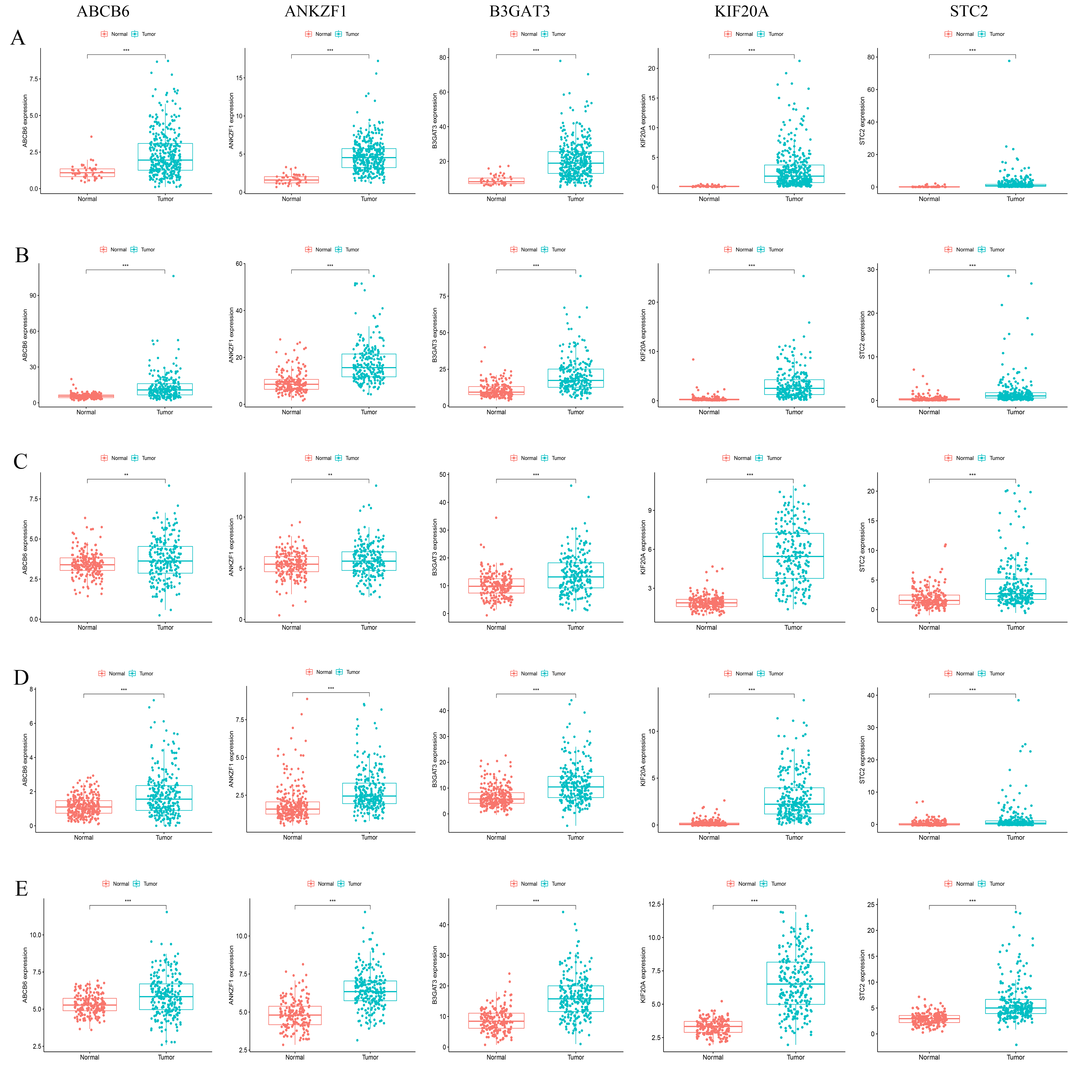

Supplement: Supplementary file 2 — Additional file 2: Figure S2. Expression validation of the five GRGs in public datasets. (A) TCGA, (B) ICGC, (C) GSE 14520, (D) GSE 25097 and (E) GSE 36376 datasets. GRGs, glycolysis-related genes; ICGC, International Cancer Genome Consortium Japan. [file 12885_2022_9209_MOESM2_ESM.tif]

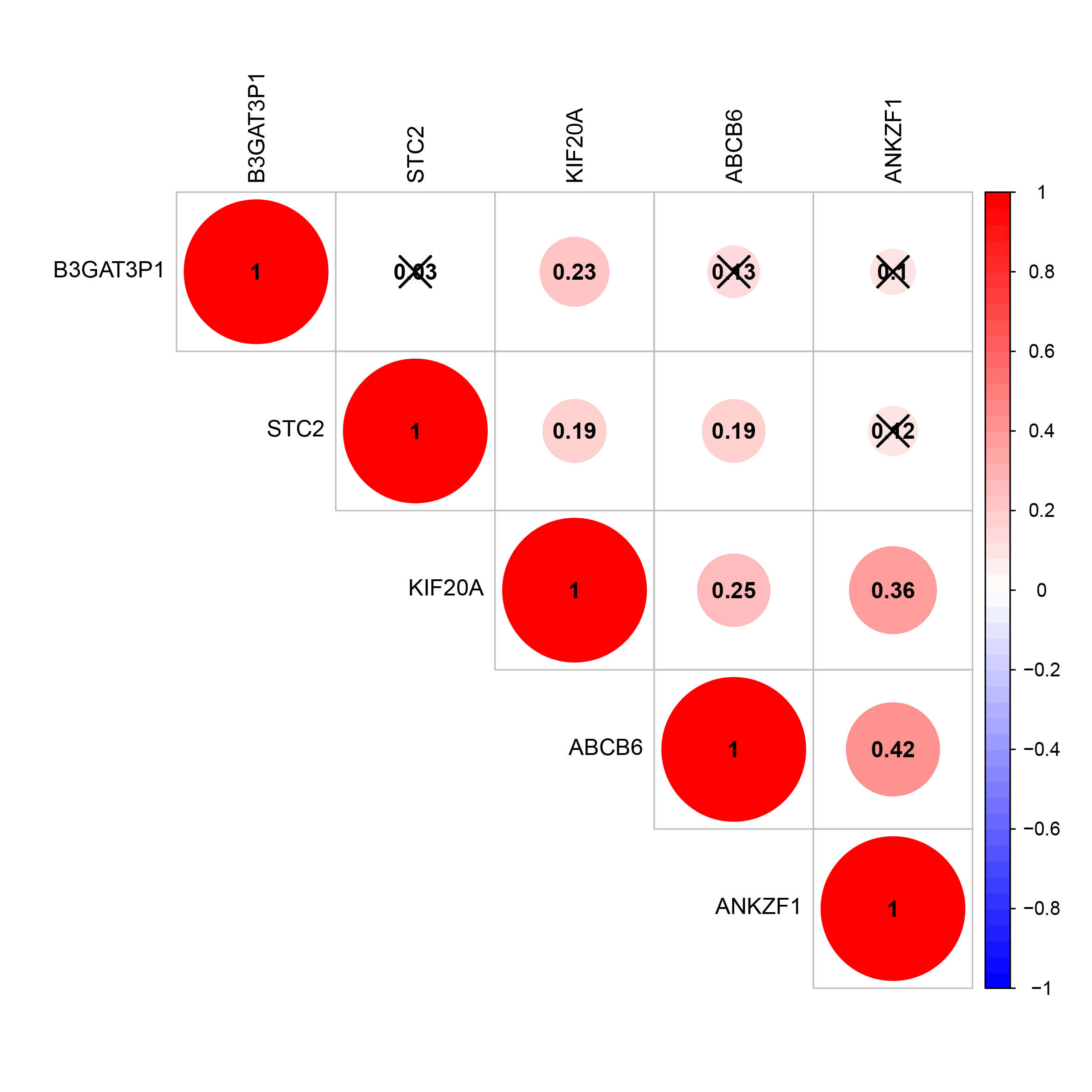

Supplement: Supplementary file 3 — Additional file 3: Figure S3. Correlation between the five glycolysis-related genes in the dataset from TCGA.) [file 12885_2022_9209_MOESM3_ESM.tif]

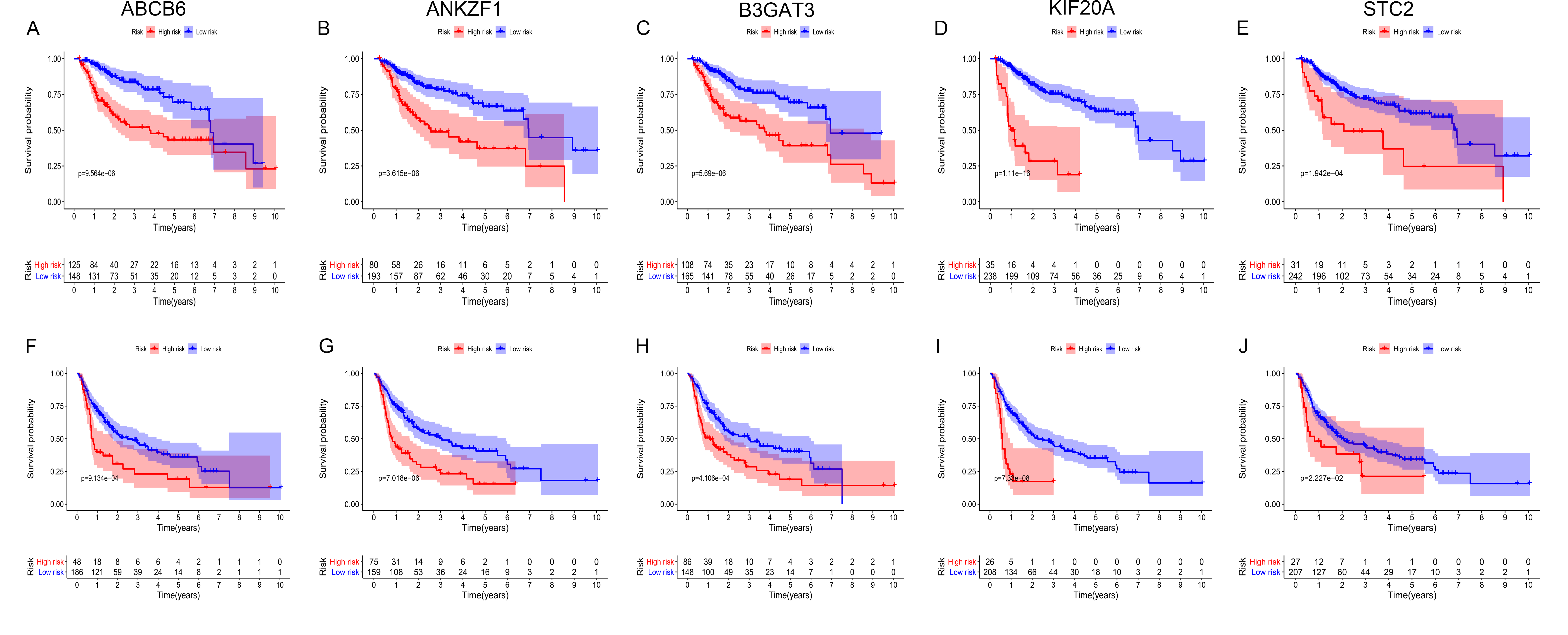

Supplement: Supplementary file 4 — Additional file 4: Figure S4. The predictive ability of the five GRGs on OS and DFS. (A-E) The relationship between the dysregulation of GRGs and OS in HCC. (F-J) The relationship between the dysregulation of GRGs and DFS in HCC. GRGs, glycolysis-related genes; HCC, hepatocellular carcinoma; OS, overall survival; DFS, disease-free survival. [file 12885_2022_9209_MOESM4_ESM.tif]
