# Supplementary material for: Identification of a glycolysis-related gene signature for predicting prognosis in patients with hepatocellular carcinoma
Source: BMC Cancer. 2022 Feb 5;22:142. doi: 10.1186/s12885-022-09209-9 (PMC8817563; doi:10.1186/s12885-022-09209-9)
Supplement: Supplementary file 5 — Additional file 5: Table S1. Detailed information for six glycolysis-related gene sets. [file 12885_2022_9209_MOESM5_ESM.docx]

Table S1 Detailed information for six glycolysis-related gene sets

| Name | Number of genes | Description | Collections |
| --- | --- | --- | --- |
| REACTOME_GLYCOLYSIS | 3 | Glycolysis Pathway | ARCHIVED C2_CP C2_CP: BIOCARTA |
| HALLMARK_GLYCOLYSIS | 200 | Genes encoding proteins involved in glycolysis and gluconeogenesis. | H |
| KEGG_GLYCOLYSIS_GLUCONEOGENESIS | 62 | Glycolysis / Gluconeogenesis | C2 CP |
| REACTOME_GLYCOLYSIS | 72 | Glycolysis | C2 CP |
| REACTOME_REGULATION_OF_GLYCOLYSIS_BY_FRU |  |  |  |
| WP_GLYCOLYSIS_AND_GLUCONEOGENESIS | 45 | Glycolysis and Gluconeogenesis | C2 CP |
