# Supplementary material for: Identification of a glycolysis-related gene signature for predicting prognosis in patients with hepatocellular carcinoma
Source: BMC Cancer. 2022 Feb 5;22:142. doi: 10.1186/s12885-022-09209-9 (PMC8817563; doi:10.1186/s12885-022-09209-9)
Supplement: Supplementary file 6 — Additional file 6: Table S2. Multivariate Cox regression analysis of GRGs independently associated with HCC prognosis. [file 12885_2022_9209_MOESM6_ESM.docx]

Table S2 Multivariate Cox regression analysis of GRGs independently associated with HCC prognosis

| Gene | coefficient | HR | 95%CI | P |
| --- | --- | --- | --- | --- |
| ABCB6 | 0.134 | 1.182 | 1.035-1.350 | 0.014 |
| ANKZF1 | 0.072 | 1.116 | 1.024-1.217 | 0.012 |
| B3GAT3 | 0.031 | 1.027 | 1.008-1.046 | 0.004 |
| G6PD | 0.001 | 1.001 | 0.993-1.009 | 0.781 |
| HMMR | 0.050 | 1.051 | 0.964-1.145 | 0.257 |
| KIF20A | 0.150 | 1.104 | 1.017-1.198 | 0.018 |
| ME1 | 0.010 | 1.010 | 0.987-1.033 | 0.409 |
| NUP85 | 0.004 | 1.004 | 0.916-1.100 | 0.940 |
| SAP30 | 0.040 | 1.041 | 0.951-1.139 | 0.388 |
| STC2 | 0.034 | 1.033 | 1.007-1.060 | 0.011 |

Note: glycolysis-related gene, GRG; hepatocellular carcinoma, HCC; hazard ratio, HR; confidence interval, CI.
