# Supplementary material for: Identification of a glycolysis-related gene signature for predicting prognosis in patients with hepatocellular carcinoma
Source: BMC Cancer. 2022 Feb 5;22:142. doi: 10.1186/s12885-022-09209-9 (PMC8817563; doi:10.1186/s12885-022-09209-9)
Supplement: Supplementary file 7 — Additional file 7: Table S3. Correlation between gene signature and clinicopathological factors. [file 12885_2022_9209_MOESM7_ESM.docx]

Table S3 Correlation between gene signature and clinicopathological factors

| Variables | N (%) | GRGs-related gene signature | | P value |
| --- | --- | --- | --- | --- |
|  |  | Low risk (%) | High risk (%) |  |
| Age (years) |  |  |  | 0.730 |
| ≤65 | 184 (67.4) | 91 (66.42) | 93 (68.38) |  |
| >65 | 89 (32.6) | 46 (33.58) | 43 (31.62) |  |
| BMI (kg/m^2^) |  |  |  | 0.570 |
| ≤24 | 129 (47.25) | 62 (45.26) | 67 (49.26) |  |
| >24 | 144 (52.75) | 75 (54.74) | 69 (50.74) |  |
| Sex |  |  |  | 0.761 |
| Female | 82 (30.04) | 40 (29.20) | 42 (30.88) |  |
| Male | 191 (69.96) | 97 (70.80) | 94 (69.12) |  |
| Race |  |  |  | 0.044 |
| White | 123 (45.05) | 70 (51.09) | 53 (38.97) |  |
| Other | 150 (54.95) | 67 (48.91) | 83 (61.03) |  |
| Vascular invasion |  |  |  | 0.019 |
| None | 152 (55.68) | 87 (63.50) | 65 (47.79) |  |
| Yes | 78 (28.57) | 35 (25.55) | 43 (31.62) |  |
| Unknown | 43 (15.75) | 15 (10.95) | 28 (20.59) |  |
| T stage |  |  |  | 0.007 |
| T1-T2 | 204 (74.73) | 112 (81.75) | 92 (67.65) |  |
| T3-T4 | 69 (25.27) | 25 (18.25) | 44 (32.35) |  |
| N stage |  |  |  | 0.541 |
| N0 | 207 (75.82) | 101 (73.72) | 106 (77.94) |  |
| N1 | 3 (1.10) | 1 (0.73) | 2 (1.47) |  |
| Unknown | 63 (23.08) | 35 (25.55) | 28 (20.59) |  |
| M stage |  |  |  | 0.020 |
| M0 | 217 (79.49) | 101 (73.72) | 116 (85.29) |  |
| M1 | 3 (1.10) | 1 (0.73) | 2 (1.47) |  |
| Unknown | 53 (19.41) | 35 (25.55) | 18 (13.24) |  |
| TNM stage |  |  |  | 0.008 |
| I-II | 202 (73.99) | 111 (81.02) | 91 (66.91) |  |
| III-IV | 71 (26.01) | 26 (18.98) | 45 (33.09) |  |
| Grade |  |  |  | <0.001 |
| I-II | 162 (59.34) | 97 (70.80) | 65 (47.79) |  |
| III-IV | 111 (40.66) | 40 (29.20) | 71 (52.21) |  |

Abbreviations: Glycolysis-related genes, GRGs; Body Mass Index, BMI.
