# Supplementary material for: Identification of a glycolysis-related gene signature for predicting prognosis in patients with hepatocellular carcinoma
Source: BMC Cancer. 2022 Feb 5;22:142. doi: 10.1186/s12885-022-09209-9 (PMC8817563; doi:10.1186/s12885-022-09209-9)
Supplement: Supplementary file 8 — Additional file 8: Table S4. Information of Immunohistochemistry in the Human Protein Atlas database. [file 12885_2022_9209_MOESM8_ESM.docx]

Table S4 Information of Immunohistochemistry in the Human Protein Atlas database

| Gene | Sample | Patient ID | Gender | Age | Staining | Intensity | Quantity | Location |
| --- | --- | --- | --- | --- | --- | --- | --- | --- |
| ABCB6 | N | 3222 | female | 63 | Not detected | Negative | None | None |
|  | T | 3196 | Male | 65 | Medium | Moderate | 75%-25% | Cytoplasmic/membranou |
| ANKZF1 | N | 3402 | Female | 54 | Low | Weak | >75% | Cytoplasmic/membranou |
|  | T | 2556 | Male | 72 | Medium | Moderate | >75% | Cytoplasmic/membranou |
| B3GAT3 | N | 3222 | female | 63 | Not detected | Negative | None | None |
|  | T | 2766 | Female | 73 | High | Strong | 75%-25% | Cytoplasmic/membranou |
| KIF20A | N | 2429 | Male | 55 | Low | Weak | >75% | Cytoplasmic/membranou |
|  | T | 3196 | Male | 65 | Medium | Moderate | >75% | Nuclear |
| STC2 | N | 2251 | Female | 50 | Not detected | Negative | None | None |
|  | T | 4969 | Male | 75 | Not detected | Weak | <25% | Cytoplasmic/membranou |
